# Supplementary material for: Film Dressings Based on Hydrogels: Simultaneous and Sustained-Release of Bioactive Compounds with Wound Healing Properties
Source: Pharmaceutics. 2019 Sep 2;11(9):447. doi: 10.3390/pharmaceutics11090447 (PMC6781310; doi:10.3390/pharmaceutics11090447)
Supplement: Supplementary file 1 [file pharmaceutics-11-00447-s001.pdf]

pharmaceutics

MDPI

# Supplementary Materials: Film Dressings Based on Hydrogels: Simultaneous and Sustained-Release of Bioactive Compounds with Wound Healing Properties

**Fabián Avila-Salas, Adolfo Marican, Soledad Pinochet, Gustavo Carreño, Oscar Valdés, Bernardo Venegas, Wendy Donoso, Gustavo Cabrera-Barjas, Sekar Vijayakumara, Esteban F. Durán-Lara**

**Table S1.** Design of PVA hydrogel nanopores (Hnp) crosslinked with different dicarboxylic acids.

| Id. | Hydrogel Nanopores (Hnp) | 2D Structure of Hydrogel Nanopores (Hnp) | 3D Structure of Hydrogel Nanopores (Hnp) |
|---|---|---|---|
| 1 | PVAnp-Oxalic acid | 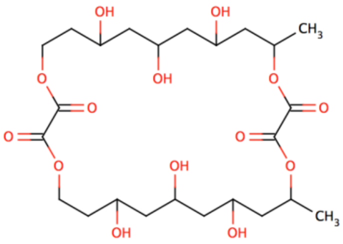 | 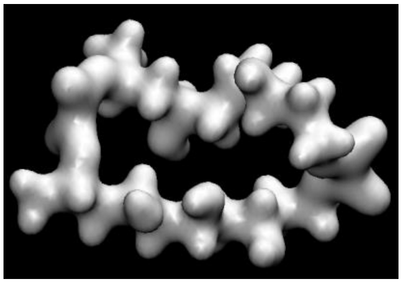 |
| 2 | PVAnp-Malonic acid | 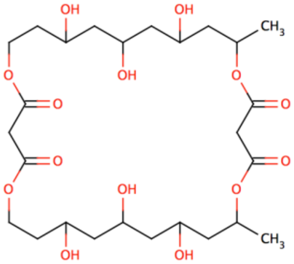 | 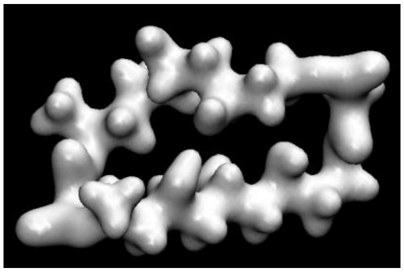 |
| 3 | PVAnp-Succinic acid | 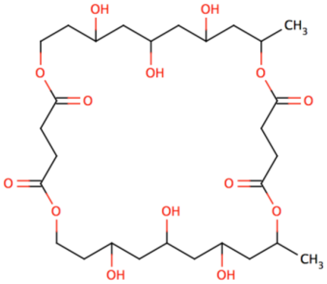 | 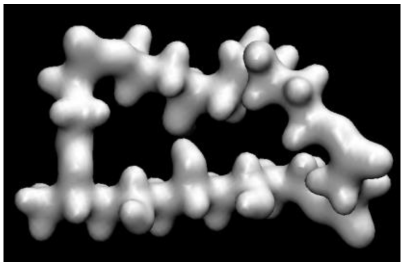 |
| 4 | PVAnp-Malic acid | 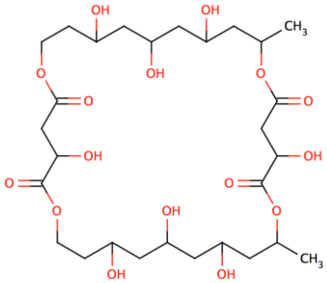 | 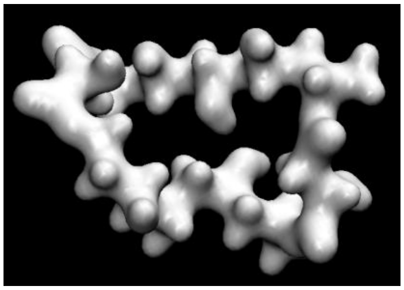 |

pharmaceutics

| | | | |
|---|---|---|---|
| 5 | PVAnp-Fumaric acid | 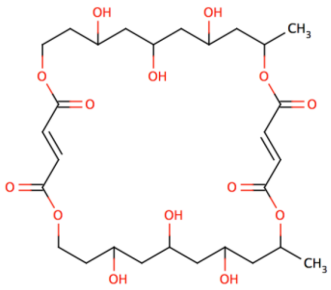 | 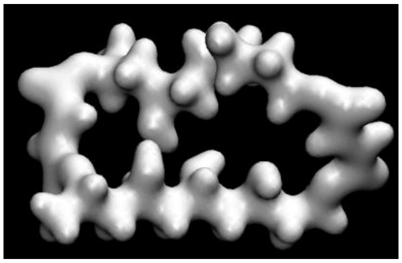 |
| 6 | PVAnp-Maleic acid | 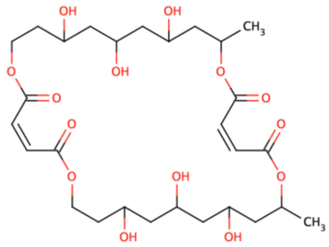 | 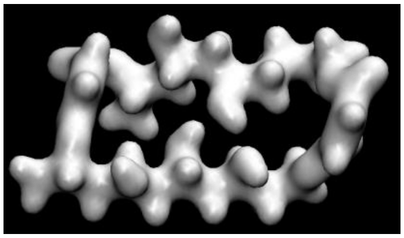 |
| 7 | PVAnp-Citraconic acid | 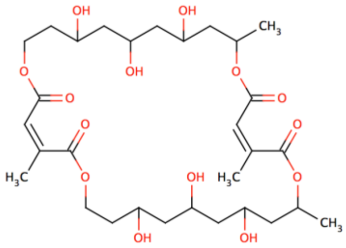 | 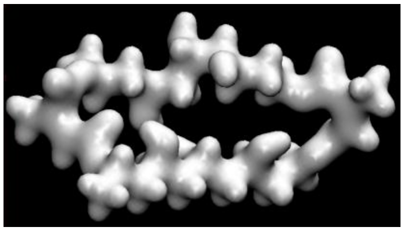 |
| 8 | PVAnp-Itaconic acid | 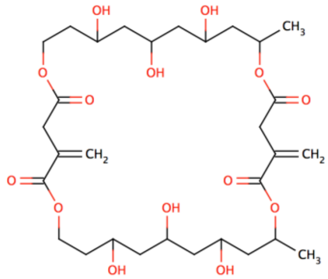 | 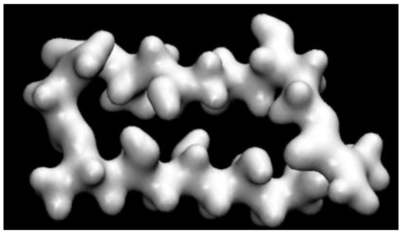 |
| 9 | PVAnp-Tartaric acid | 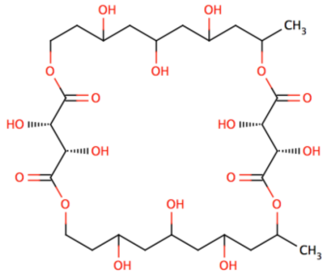 | 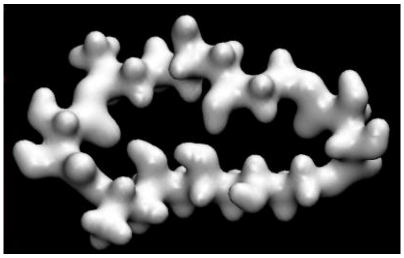 |
| 10 | PVAnp-Glutaric acid | 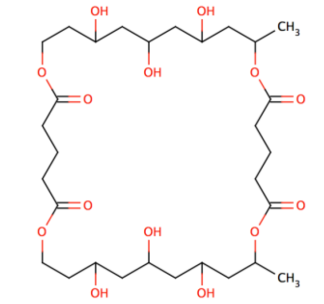 | 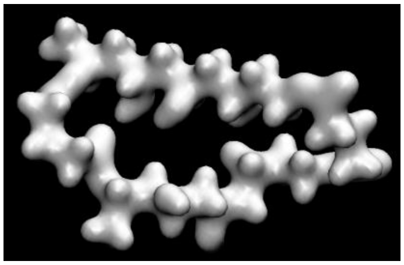 |

pharmaceutics

| | | | |
|---|---|---|---|
| 11 | PVAnp-Adipic acid | 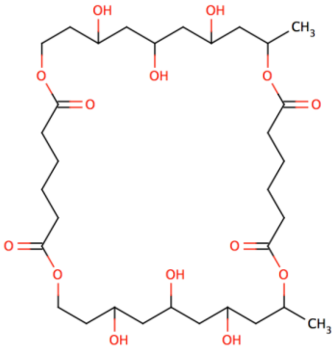 | 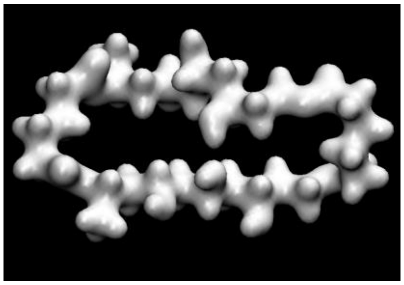 |
| 12 | PVAnp-Pimelic acid | 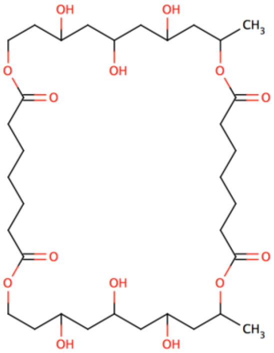 | 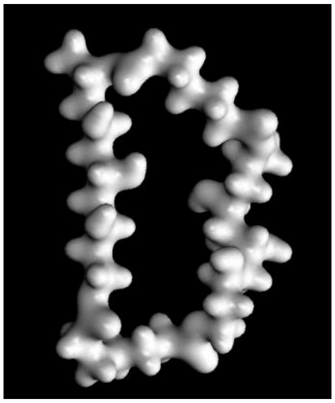 |
| 13 | PVAnp-Suberic acid | 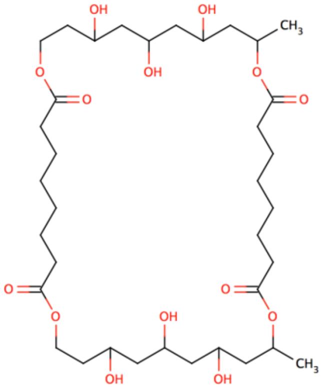 | 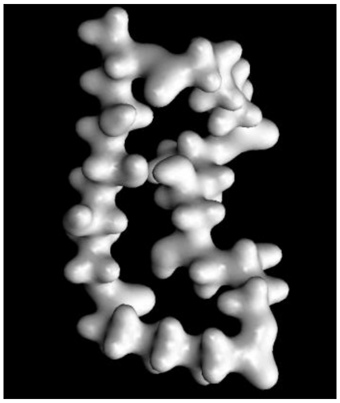 |
| 14 | PVAnp-Azelaic acid | 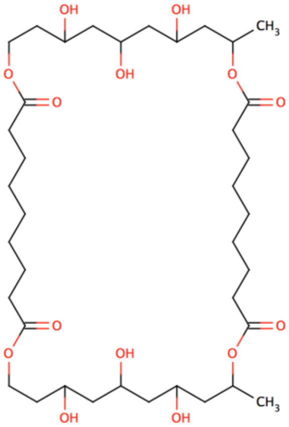 | 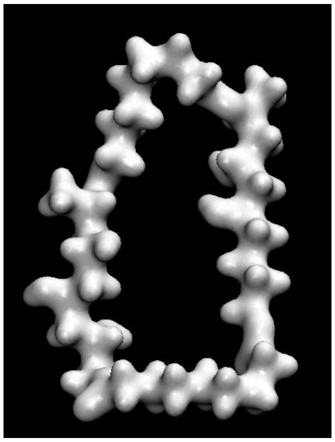 |

| | | | |
|---|---|---|---|
| 15 | PVAnp-Phtalic acid | 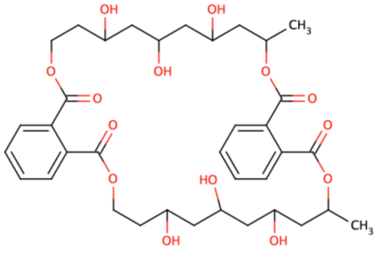 | 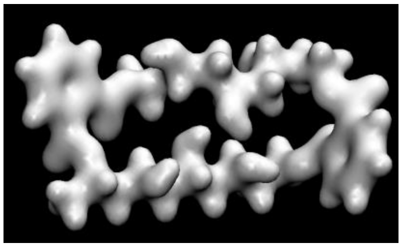 |
| 16 | PVAnp-Isophtalic acid | 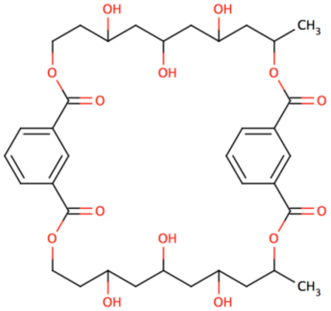 | 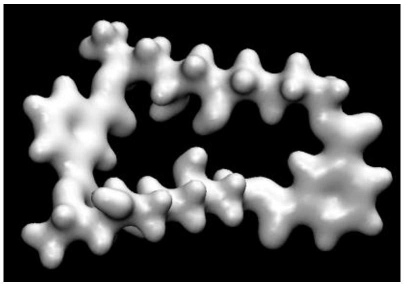 |
| 17 | PVAnp-Terephtalic acid | 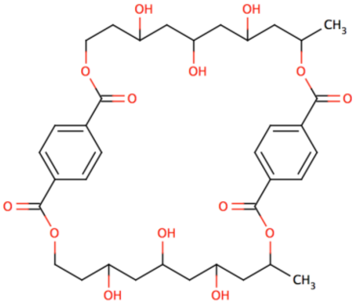 | 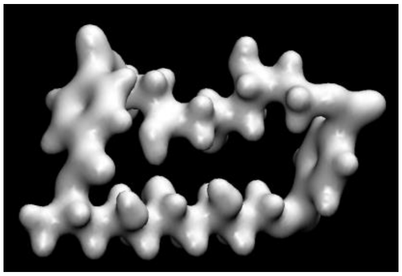 |
| 18 | PVAnp-2,5-pyridin acid | 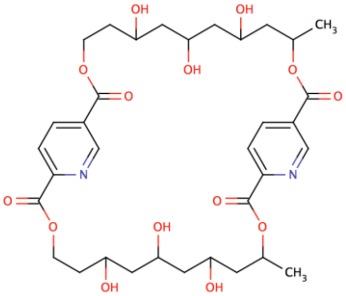 | 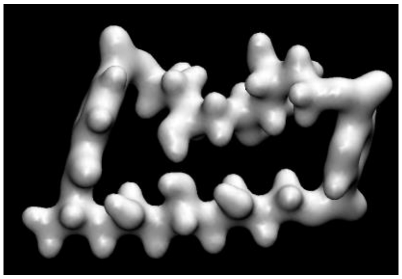 |
| 19 | PVAnp-Aspartic acid | 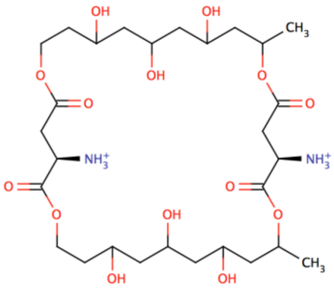 | 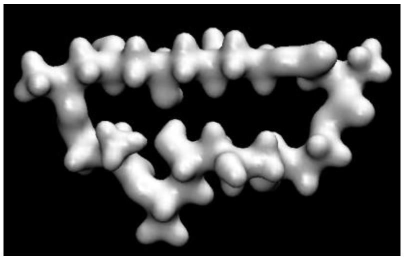 |

| | | | |
|---|---|---|---|
| 20 | PVAnp-Glutamic acid | 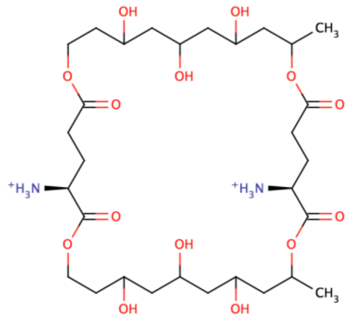 | 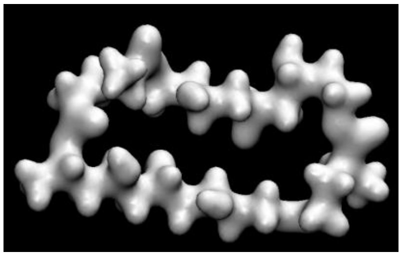 |



**Table S2**. Vibration modes and band frequencies of PVA and PDCAH.

| Identification | Chemical group | | Wave numbers ($cm^{-1}$) |
|---|---|---|---|
| | PVA<br>PSAH<br>PMALiH<br>PAAH<br>PMALEH | O-H from the intermolecular and intramolecular hydrogen bonds | ν ~3400 |
| | PAAH | N-H from alkyl-$NH_2$ group | ν ~3400 (overlapping whit the OH bands from PVA) |
| | PMALEH | O-H from maleic acid | ν 3380 |
| | PVA<br>PSAH<br>PMALiH<br>PAAH<br>PMALEH | C-H from alkyl groups | ν 2840-3000 |
| | PSAH | C=O | ν 1704 |
| | PMALiH | | ν 1715 |
| | PAAH | | ν 1689 |
| | PMALEH | | ν 1697 |
| | PAAH | CO-NH | ν 1630 and 1419 |
| | PVA<br>PMALiH | -C=C | ν 1640<br>ν 1636 |
| | PMALEH | CO-CH=CH | ν 1627 |
| | PMALiH | C-O | δ 1180 |
| | PVA | CO (crystallinity) | ν 1100 |
| | PVA<br>PSAH<br>PMALiH<br>PAAH<br>PMALEH | C-O-C | ν 1150-1085 |
| | PVA<br>PSAH<br>PMALiH<br>PAAH<br>PMALEH | $CH_2$ | δ 1461-1417 |

pharmaceutics

MDPI

**Table S3**. Mechanical parameters of **PDCAH**.

| PDCAH | σm, MPa | εB, % | E, MPa |
|---|---|---|---|
| **PMALEH** | 13.1 | 183.9 | 61.1 |
| **PAAH** | 9.5 | 37.8 | 176.4 |
| **PSAH** | 19.3 | 220.7 | 82.4 |
| **PMALIH** | 17.4 | 209.9 | 63.8 |

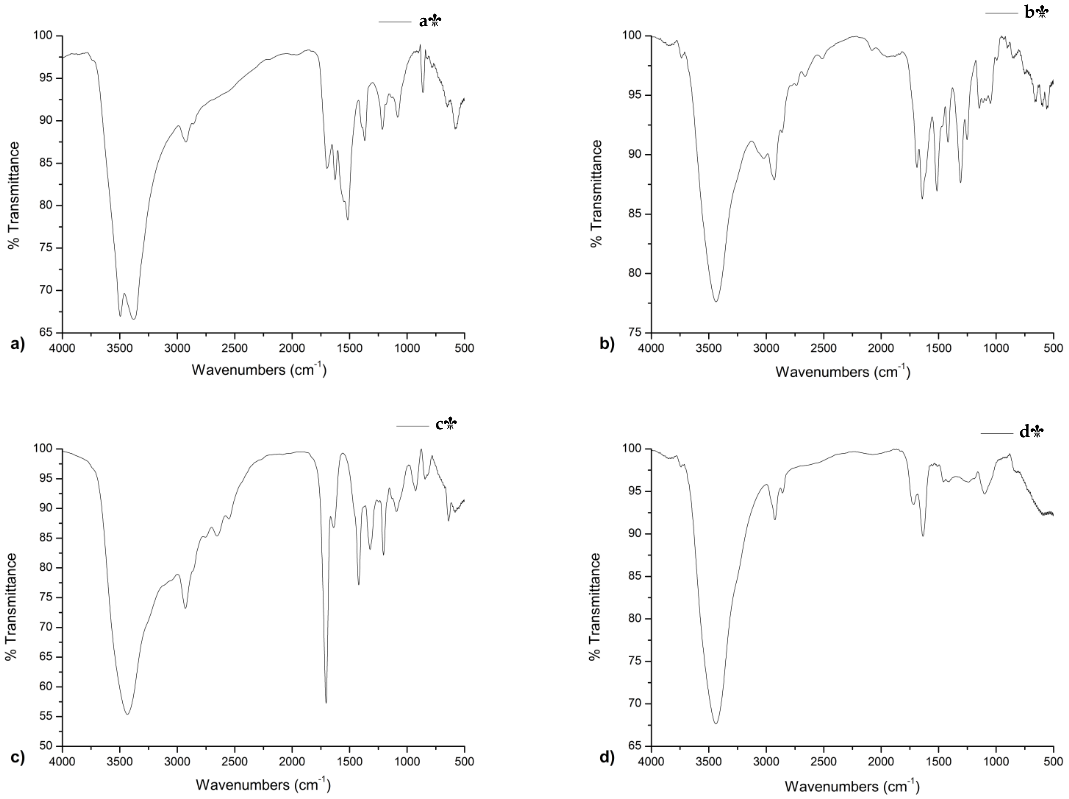


**Figure S1**. FTIR spectra of (**a**) PMALEH; (**b**) PAAH; (**c**) PSAH and (**d**) PMALIH.
